# A meta-analysis of cognitive and functional outcomes in severe brain trauma cases

**DOI:** 10.3389/fnbeh.2024.1349672

**Published:** 2024-03-14

**Authors:** Zhang Shuanglong, Yuan Jiangyuan, Nie Meng, Wang Zheng, Zhang Yunshui, Sun Wei, Qiao Li, Jiang Rongcai

**Affiliations:** ^1^Department of Neurosurgery, Tianjin Medical University General Hospital, Tianjin, China; ^2^Department of Critical Care Medicine, Peking University International Hospital, Beijing, China; ^3^Department of Critical Care Medicine, The Air Force Characteristic Medical Center, Air Force Medical University, Beijing, China

**Keywords:** brain trauma, severe TBI, depression, verbal and visual disabilities, learning and memory impairment, cognitive disabilities

## Abstract

**Background:**

Severe traumatic brain injuries (TBIs) are an important health issue worldwide, which are associated with harmful side effects. This meta-analysis investigates the cognitive and functional outcomes in severe brain trauma cases. It assesses the impact on memory, verbal and visual abilities, attention, learning, and the presence of depression. The study provides a comprehensive overview of the consequences of severe brain trauma injury on cognitive and functional domains.

**Objective:**

The main objective of the current comprehensive meta-analysis study is to assess and analyze the impact of severe TBI on functional and cognitive outcomes, including verbal, visual, attention, learning, memory, and emotional stability.

**Methods:**

We collected data from three online databases, including PubMed, Cochrane Library, and Embase. Case–control trials related to severe TBI association with cognitive and functional outcomes were included. Verbal strength, visual functions, learning abilities, attention, memory, and depression were considered primary outcomes.

**Results:**

We have included 13 case–control studies with 1,442 subjects in this meta-analysis, which provide adequate data to determine the pooled effect size for targeted outcomes. The effect of severe TBI on the inducement of depression and impairment of memory, verbal, visual, attention, and learning abilities compared to the control group showed statistically significant outcomes (*p* < 0.05).

**Conclusion:**

Severe TBI is strongly associated with impaired cognitive and functional abilities, including visual and verbal disabilities, impaired memory, depression inducement, attention deficits, and learning disabilities.

## 1 Introduction

Severe traumatic brain injury (TBI) represents a major global health concern, with profound implications for individuals, families, and healthcare systems. Survivors of severe brain trauma often face long and arduous journeys toward recovery, marked by a spectrum of cognitive and functional challenges. Understanding the factors that influence cognitive and functional outcomes in this population is not only crucial for optimizing patient care but also for informing healthcare policies and interventions (Robinson, 2021).

The pathophysiology of severe brain trauma is complex and multifaceted. The initial mechanical insult, often resulting from accidents, falls, or violence, sets in motion a cascade of events, including primary and secondary injury mechanisms that can lead to structural damage, inflammation, and neurochemical imbalances (Orr and Gensel, 2017; Ng and Lee, 2019). These processes, occurring within the highly delicate and intricately interconnected neural networks of the brain, can result in a wide range of neurological deficits (Ng and Lee, 2019). While advances in neuroimaging and neurocritical care have expanded our understanding of TBI, there remains a pressing need to comprehensively assess the cognitive and functional consequences of severe brain trauma (Pavlovic et al., 2019; Olsen et al., 2021; Howlett et al., 2022). Various case–control research studies have concluded that the incidence of severe TBI is strongly linked to altered cognitive functionality. As a consequence of the brain injury, TBI patients experienced impaired memory and altered verbal, visual, and learning functions. Moreover, the prevalence of depression is significantly higher in severe TBI cases (Hellawell et al., 1999; Ashman et al., 2008).

Our objective is to provide a comprehensive and evidence-based understanding of the cognitive and functional outcomes in severe TBI cases. This study represents an extensive exploration of cognitive and functional outcomes in severe brain trauma cases through a comprehensive meta-analysis. Our analysis synthesizes data on a range of cognitive domains, including memory, attention, vision, language, and executive functions. By synthesizing data on functional outcomes, we aim to highlight the challenges faced by TBI survivors and the target areas where intervention strategies may be most effective.

## 2 Materials and methods

### 2.1 Search methodology

Various search engines were employed to gather data, including the Cochrane Library,^1^ PubMed,^2^ and Embase^3^ databases. We used Medical Subject Headings (MeSH) terms as keywords, including “traumatic brain injury,” “severe TBI,” “cognitive impairments associated with TBI,” “severe TBI related physical impairments,” and “TBI induced depression.” Moreover, aside from conducting a methodical search on PubMed, we expanded our search scope by utilizing the “related articles” feature to precisely evaluate the abstracts, studies, and citations. Our database search was confined to records available exclusively in the English language.

### 2.2 Selection standards for included studies

This comprehensive meta-analysis has included case–control studies aimed at assessing the association between severe TBI and the development of cognitive disorders and physical impairment. The study included published data spanning various time periods from 1997 to the present, to ensure thorough coverage of the existing literature. In order to maintain the integrity of the exclusion criteria, the following aspects were considered in study selection: (1) the exclusion of animal studies, (2) only limited to studies with published data, (3) the exclusion of articles with inappropriate methodology, data collection, statistical analysis, and overall outcome quality, (4) avoidance of derivative data sources, such as review articles, (5) the exclusion of participants with severe TBI who also had concurrent life-threatening conditions such as cancer, cardiac disorders, neurodegenerative disorders, and other neurological conditions., which ensured a focused analysis of the specific impact of TBI on health outcomes, and (6) the exclusion of participants with mild and moderate TBI.

The characteristics of all included studies are concisely summarized in Table 1. For data retrieval and inclusion, the following parameters were taken into account: (1) case–control studies were selected to gather data on the association between severe TBI and cognitive disorders and physical impairments, (2) the focus was exclusively on patients with severe TBI, (3) studies with sufficient sample size, (4) inclusion of studies with verified measurement methods and appropriate statistical analyses, (5) population inclusion of both males and females, and (6) the inclusion of research studies with well-conducted analyses and outcomes.

**Table 1:** Characteristics and findings of the included studies.

Sr. no.	Author/country	Year	Study design	Study participants	Sex	Age	Follow-up period	Outcome measures	Evaluation	References
1	Ashman et al.,/USA	2008	Case–control study	54 TBI patients and 40 control	Both males and females	> 55	6 months to 1 year	CVLT, WMS-LM, WMS-VR, WMS-LM, WMS-LM, COWAT (FAS), WCST CS	TBI patients have impaired attention and verbal memory	Ashman et al. (2008)
2	Peretz et al.,/Israel	1997	Case–control study	54 TBI patients and 10 control	Both males and females	> 60	-	WMS-VR	In older TBI patients, impaired visual activity has been found	Aharon-Peretz et al. (1997)
3	Breed et al.,/USA	2008	Case–control study	56 TBI patients and 50 control	Both males and females	> 65	-	WMS-VR, WMS-LM	In older TBI patients, cognitive impairment has been found	Breed et al. (2008)
4	Rapoport et al.,/Canada	2006	Case–control study	49 TBI patients and 69 control	Both males and females	> 50	1 year	COWAT, CVLT	With aging, in TBI patients, cognitive functions significantly declined	Rapoport et al. (2006)
5	Rao et al.,/USA	2010	Case–control study	The total number of participants was 43	Both males and females	> 45	1 year	MMSE	In the early-aged TBI patients, mild depression has been evident	Rao et al. (2010)
6	Ping ma et al.,/Taiwan	2019	Case–control study	The total number of participants was 440	Both males and females	> 45	2 year	PSQI, BDI, BAI	In the TBI patients, sleep disorder, anxiety, and depression have been evident	Ma et al. (2019)
7	Richard et al.,/Canada	2000	Case–control study	The total number of participants was 80	Both males and females	> 26	-	BDI, CVLT	Anxiety and learning difficulties have been found to be more significant in elderly TBI patients	Richards (2000)
8	Hellawell et al.,/UK	1999	Retrospective case–control study	The total number of participants was 96	Both males and females	> 25	1 year	NART, TMT, GOS, HISC	Patients with moderate to severe brain injuries have experienced altered physical and cognitive functions	Hellawell et al. (1999)
9	Sigurdardottir et al.,/Norway	2009	Case–control study	The total number of participants was 115, where 41 cases had severe brain injuries	Both males and females	> 25	1 year	GOSE, COWAT	In severe TBI patients, cognitive recovery after 1 year of follow-up was found insignificant	Sigurdardottir et al. (2009)
10	Salmond et al.,/UK	2005	Case–control study	The total number of participants was 63, where 31 cases had severe brain injuries and 32 were control	Both males and females	> 30	-	BDI	The results revealed that TBI patients experienced severe depression	Salmond et al. (2005)
11	Anderson et al.,/Australia	2004	Case–control study	The total number of participants was 117, where 84 cases and 33 control	Both males and females	2–7	-	EOWPVT, NMI,	Learning and memory disabilities have been significantly found in TBI patients compared to healthy individuals	Anderson et al. (2004)
12	Hawley et al.,/UK	2004	Case–control study	The total number of participants was 81, where 67 cases and 14 control	Both males and females	6–18	-	WISC-III	In the TBI patients, intellectual and verbal strengths were found compromised	Hawley (2004)
13	Mangels et al.,/USA	2002	Case–control study	The total number of participants was 25, where 15 cases and 10 control	Both males and females	> 25	-	WMS*-*R	Significant evidence of impaired memory has been observed in severe TBI patients	Mangels et al. (2002)

### 2.3 Extraction of data

**Figure 1:**
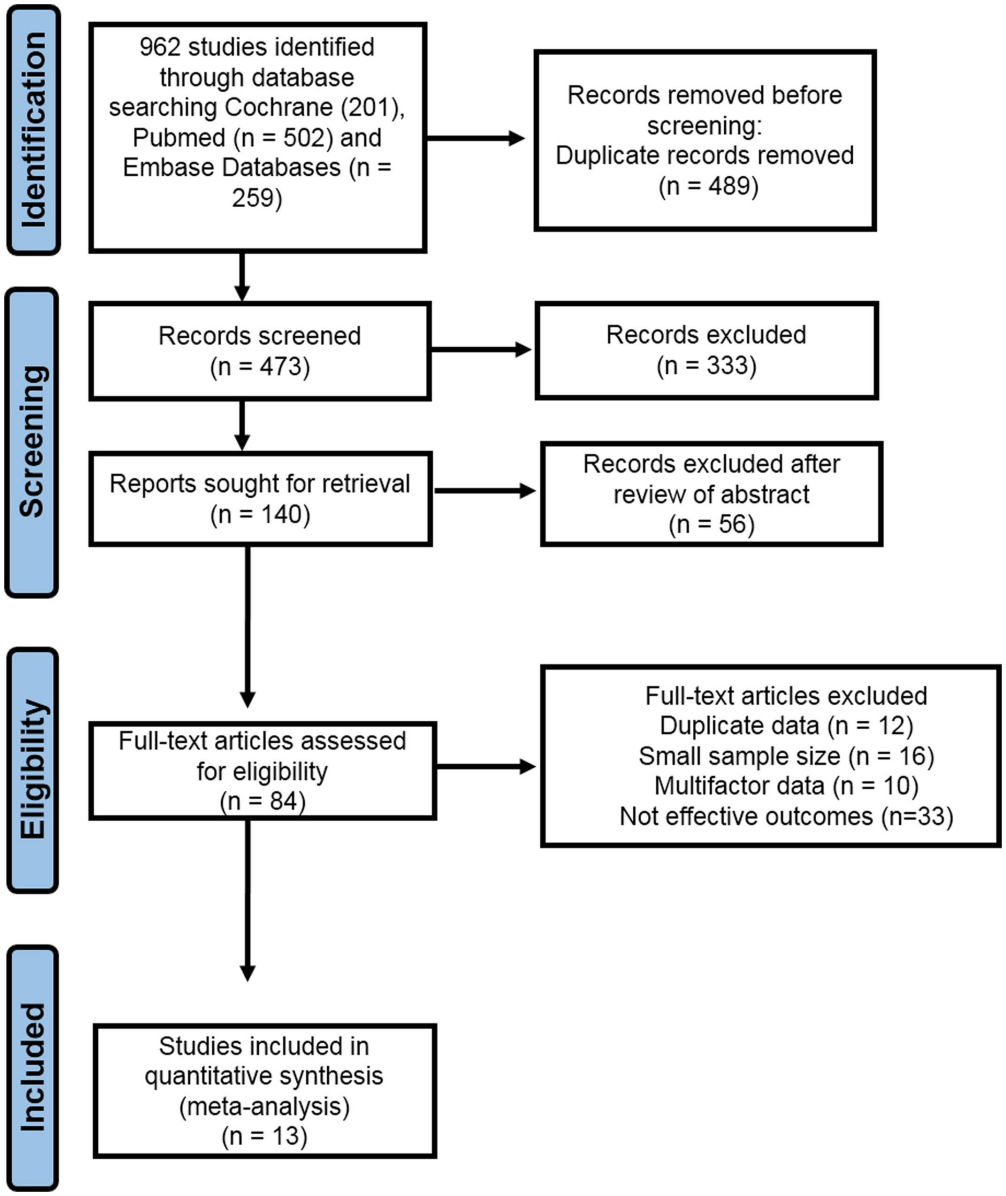
A visual representation of the included studies via systemic PRISMA flow chart.

Two investigators independently conducted data extraction. Data from the studies included in the analysis were systematically collected and organized within a standardized Excel spreadsheet. The collected data from these studies have included various information, including author names, study locations, publication years, sample sizes, sex distribution, age demographics, follow-up periods, outcome measurements, and research findings. The study selection process is shown in Figure 1, using the PRISMA flow chart. We extracted mean values, standard deviations, and sample sizes from the research studies to evaluate the potential risk of cognitive disorders and physical impairments associated with severe TBI.

To create a comprehensive database for our meta-analysis, a Microsoft Excel spreadsheet was established, including all the pertinent data for the analysis, and was thoroughly reviewed by the third author to resolve any inconsistencies. The third author precisely addressed discrepancies through comprehensive data examination, consultations with co-authors, and utilization of statistical methodologies, ensuring a robust and reliable meta-analysis outcome.

### 2.4 Description of included studies

We have included 13 case–control studies in this meta-analysis. In total, 6 studies out of 13 have a population age of ≥45 years and 7 studies include patient populations of age ≥ 25 years. In all included studies, the risk of cognitive disorders, including depression, impaired memory, and poor attention, was assessed along with the physical impairments such as visual disabilities and impaired verbal and learning functions in severely traumatic brain-injured patients. In Table 1, the characteristics and findings of the included studies are summarized.

Following outcome measures from the case–control studies were involved in the pooled estimation of the analysis, such as CVLT, WMS-III, WMS-LM, ES, WMS-VR, COWAT (FAS), WCST CS, MMSE, PSQI, BAI, BDI, GOS, NART, TMT, HISC, GOSE, EOWPVT, NMI, and WMS*-*R (Table 2). The current comprehensive meta-analysis was performed, involving a meticulous literature search that identified and included 13 research studies. In the first step, a total of 962 studies were identified via database search. After removing duplicates and conducting a preliminary screening of full-text articles, a total of 84 articles were assessed for eligibility. Out of these, 71 studies were excluded based on criteria such as duplicate data, limited sample size, unreliable and complex data, poor assessment methodology, and sub-optimal outcomes. The study selection is illustrated in a PRISMA flow diagram (Figure 1).

**Table 2:** List of abbreviations with full forms.

Sr. no.	Abbreviations	Full form
1	CVLT	California verbal learning test
2	WMS-III	Wechsler memory scale—third edition
3	WMS-LM	Wechsler memory scale—logical memory
4	WMS-ES	Wechsler memory scale—effect size
5	VR	Visual reproduction
6	COWAT (FAS)	Controlled oral word association test (FAS)
7	WCST CS	Wisconsin card sorting test—categories solved
8	MMSE	Mini-mental state examination
9	PSQI	Pittsburgh sleep quality index
10	BAI	Beck anxiety inventory
11	BDI	Beck depression inventory
12	GOS	Glasgow outcome scale
13	NART	National adult reading test
14	TMT	Trail making test
15	HISC	Hopkins verbal learning test—immediate recall
16	GOSE	Glasgow outcome scale—extended
17	EOWPVT	Expressive one-word picture vocabulary test
18	NMI	Neuropsychological memory index
19	WMS*-*R	Wechsler memory scale—revised
20	SMD	Standardized mean difference

### 2.5 Statistical analyses

In this meta-analysis, all statistical analyses were performed using Review Manager, Version 5.3 (Cochrane Collaboration in Oxford, England). The study adhered to the Preferred Reporting Items for Systematic Reviews and Meta-Analyses (PRISMA) guidelines. The means and standard deviations of included research studies were extracted to determine the association between severe TBI and the risk of cognitive disorders and physical impairments by a random-effects model. The estimated pooled mean difference and the corresponding 95% confidence interval (CI) were calculated to estimate the incidence of cognitive disorders in severe TBI patients. We assessed heterogeneity among the studies via the chi-square (χ2) test and the I^2^ statistics. Cochran’s Q-test yielded a *p*-value below 0.10, indicating significant statistical heterogeneity.

## 3 Results

### 3.1 Association between severe TBI and verbal, visual, and learning abilities

**Figure 2:**
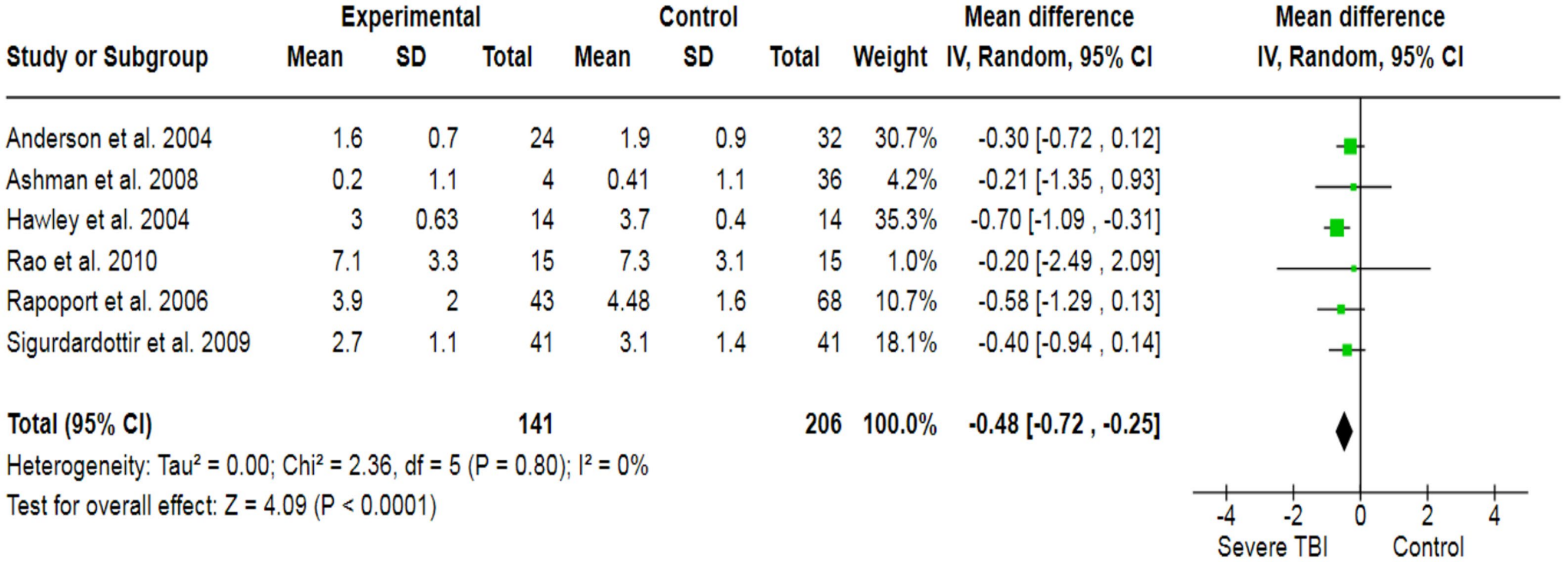
The forest plot of severe TBI and the risk of verbal impairment; diamond (pooled estimate), squares (individual study effects), and horizontal lines (confidence intervals).

**Figure 3:**
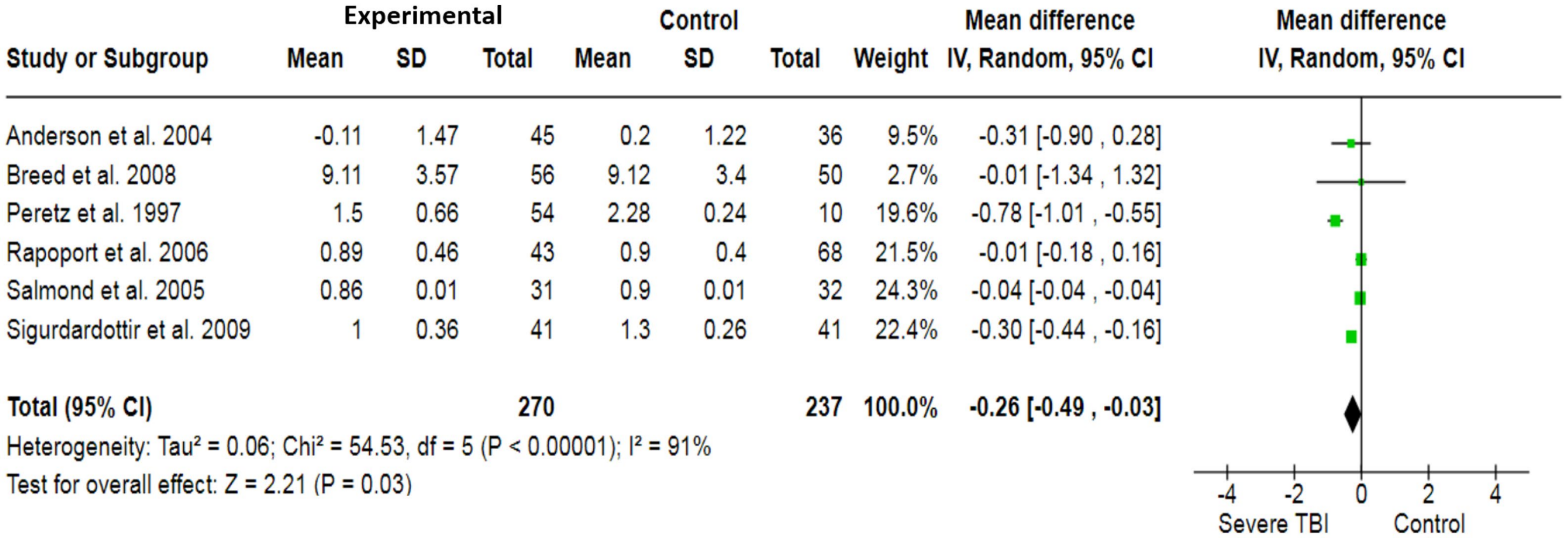
The forest plot of severe TBI and visual impairment risk; diamond (pooled estimate), squares (individual study effects), and horizontal lines (confidence intervals).

**Figure 4:**
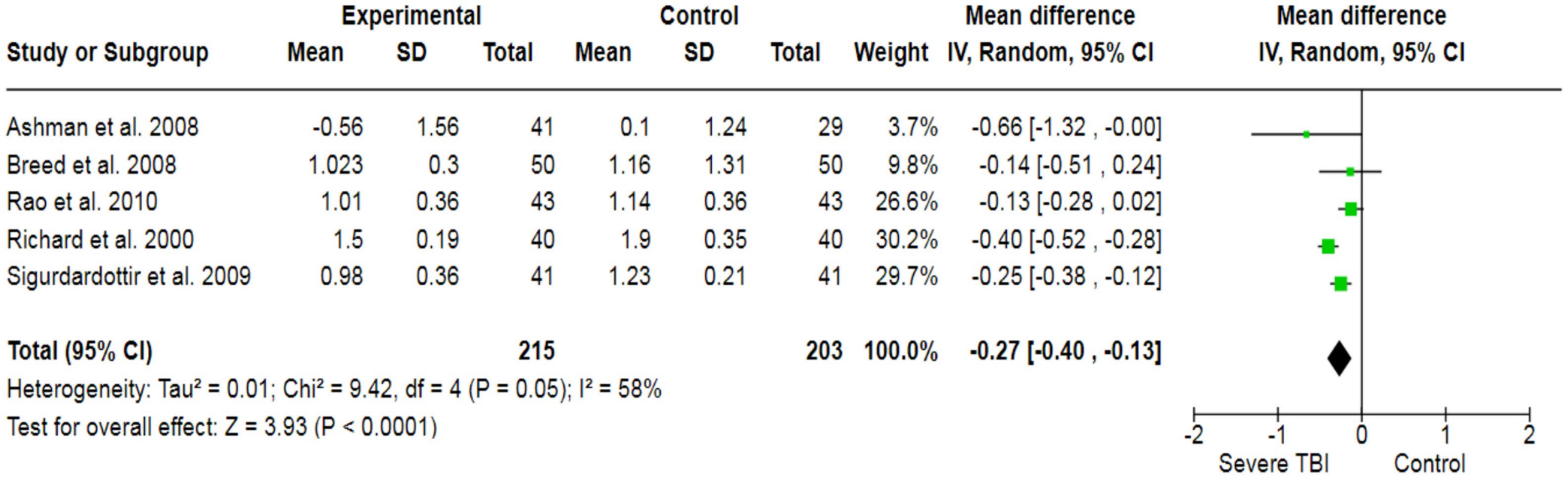
The forest plot of severe TBI and the risk of impairment of learning abilities; diamond (pooled estimate), squares (individual study effects), and horizontal lines (confidence intervals).

To assess the association between severe TBI and verbal functionality, a total of 6 case–control studies with a sample size of 568 have generated a pooled estimate of the study’s effect size (Figure 2). The outcomes have reported an SMD of −0.48, which suggests a substantial effect size, indicating that individuals with severe TBI possess lower verbal performance compared to healthy individuals. For visual quality, a total of 6 studies (507 participants) have produced a pooled SMD of −0.26 using a random-effect model, showing a strong association of severe TBI with poor visual outcomes (Figure 3). Moreover, 5 case–control trials with 418 subjects have produced a pooled SMD of −0.27, indicating altered learning abilities in severe TBI patients (Figure 4).

### 3.2 Association between severe TBI and risk depression, altered memory, and impaired attention

**Figure 5:**
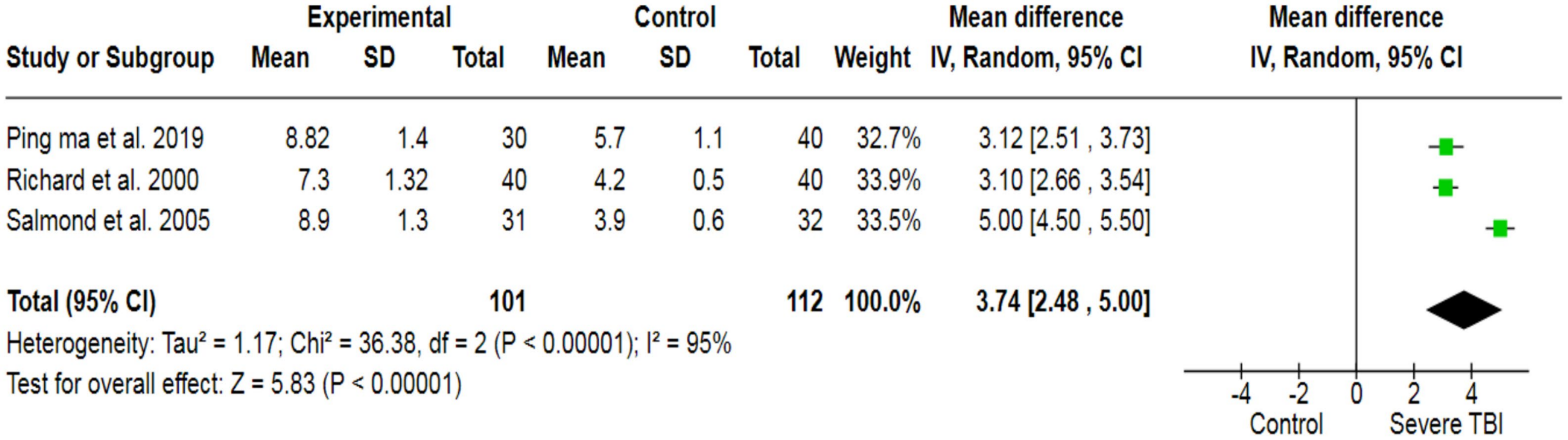
The forest plot of severe TBI and depression risk; diamond (pooled estimate), squares (individual study effects), and horizontal lines (confidence intervals).

**Figure 6:**
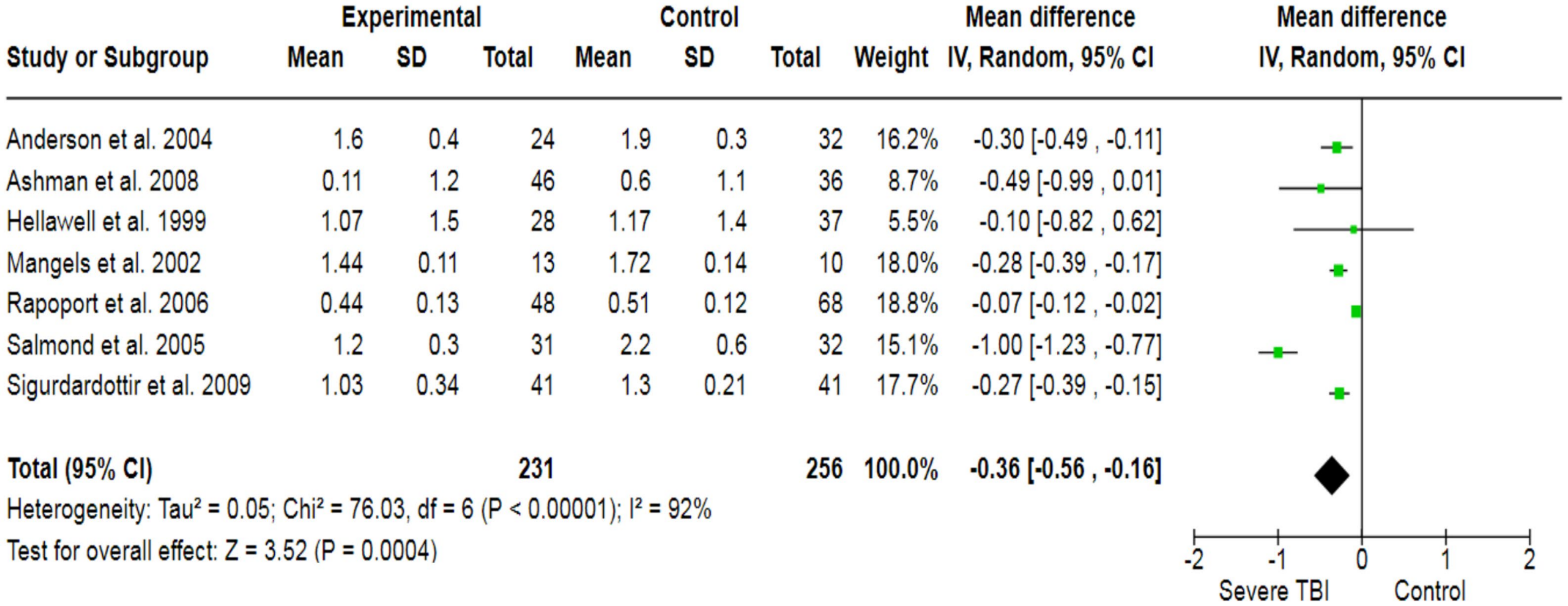
The forest plot of severe TBI and the risk of altered memory; diamond (pooled estimate), squares (individual study effects), and horizontal lines (confidence intervals).

**Figure 7:**
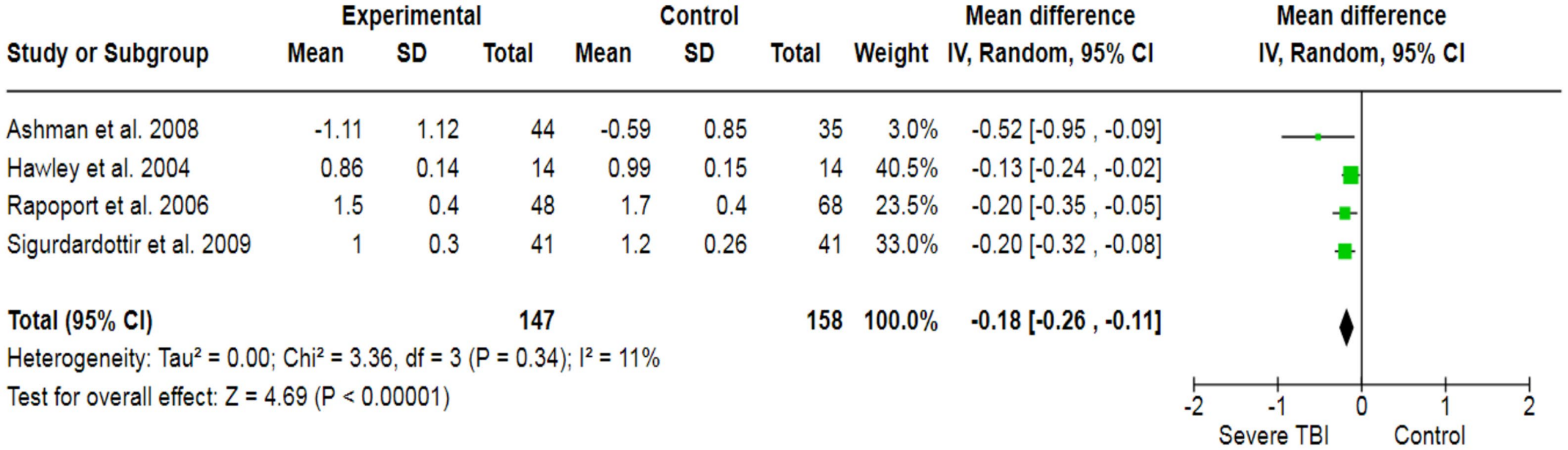
The forest plot of severe TBI and the risk of altered attention; diamond (pooled estimate), squares (individual study effects), and horizontal lines (confidence intervals).

To assess the potential inducement of depression in severe TBI patients, we analyzed data from three studies (213 participants), which collectively yielded a pooled effect size of SMD = 3.74 (Figure 5). This indicated a significant association between severe TBI and an increased likelihood of experiencing depression compared to healthy individuals. Additionally, our analysis revealed a strong association between severe TBI and the risk of altered memory (pooled SMD: −0.36) (Figure 6), as well as a significant association between severe TBI and impaired attention (pooled SMD: −0.18) (Figure 7).

### 3.3 Heterogeneity and a risk of bias

The heterogeneity test was statistically analyzed via the Cochran Q-test. A large heterogeneity was found among studies concerning the relationship between severe TBI and the risk of visual impairment (I^2^ = 91%), learning abilities (I^2^ = 58%), the risk of depression (I^2^ = 95%), altered memory (I^2^ = 92%), and impaired attention (I^2^ = 58%), which may have influenced the validity and generalizability of the results.

**Figure 8:**
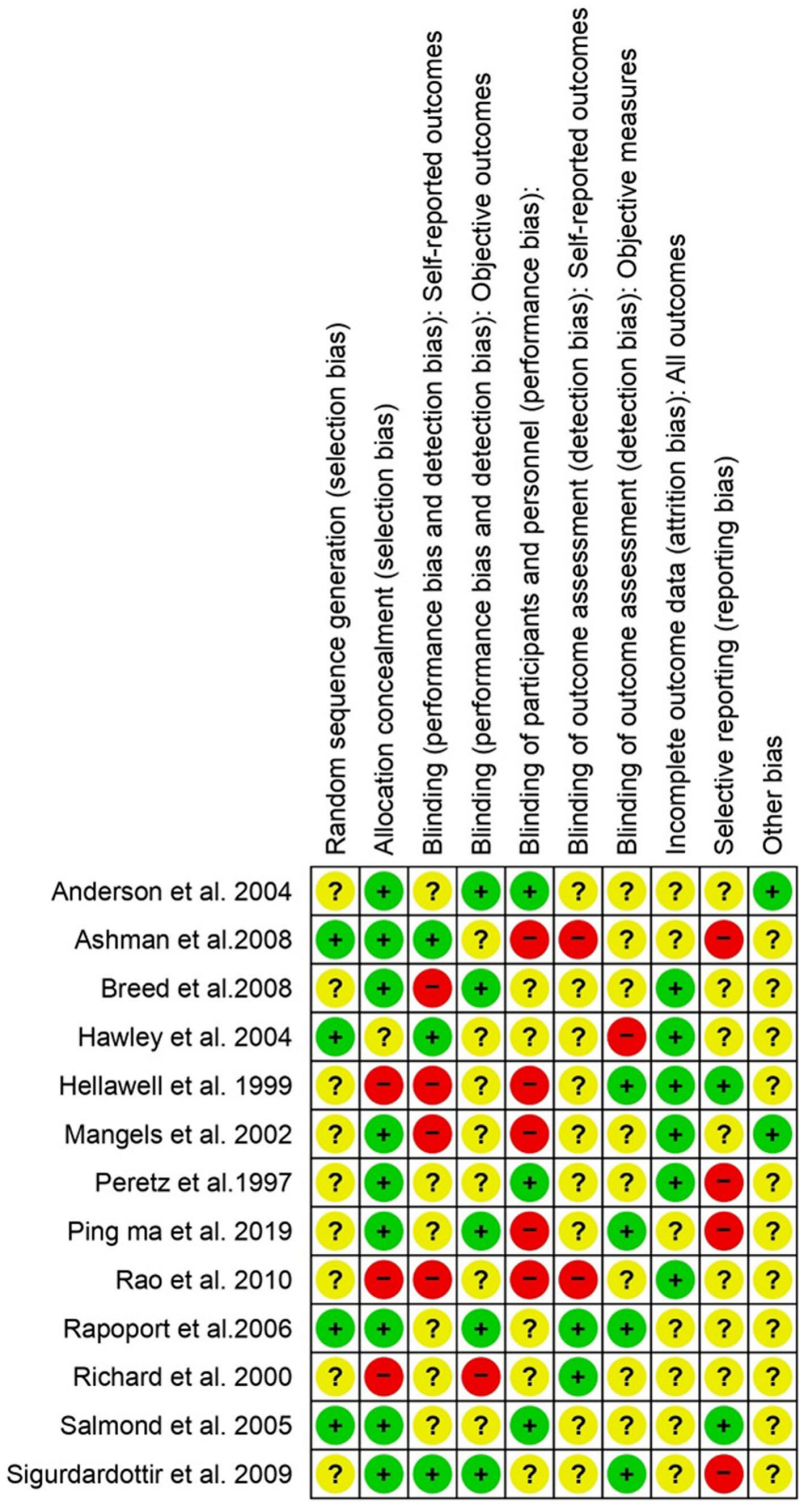
The risk of bias assessment for each included study in accordance with “Cochrane RoB 2”. Low risk (+), unclear (?), and high risk (−).

The potential risk of bias for each included study was independently evaluated by the Cochrane Collaboration to assess methodological quality (Figure 8). Several research trials showed high risk, with major sources of bias associated with performance bias, allocation concealment, self-reporting outcomes, random sequence generation, and protection from other potential biases. Due to the challenge of unsegregated participants and the age factor, along with multiple outcome measures, the majority of RCTs included here are at greater risk of bias.

## 4 Discussion

Severe TBI significantly affects various dimensions, including cognitive functions, emotional wellbeing, and physical abilities. The complex interplay of neural damage, disrupted neurochemical balance, and associated complications often leads to critical challenges in memory and executive functions, emotional regulation, and motor skills. Additionally, it can lead to behavioral changes and impaired learning abilities. These alterations disturb a person’s social and psychological wellbeing (Arciniegas et al., 2002). The primary aim of this comprehensive meta-analysis is to determine the altered functional cognitive impairments, including verbal, visual, learning, attention, and memory disabilities, and the inducement of depression in severe traumatic brain-injured patients. This comprehensive meta-analysis review included 13 studies with 1,442 study participants, which provided data for analysis. The estimated pooled analysis outcomes of our study revealed that severe traumatic brain injuries have a significantly strong association with impaired memory, visual functionality, learning abilities, verbal functions, attention deficits, and the inducement of depression.

Numerous research studies corroborate our findings. An investigative study has shown that patients who have experienced severe TBI exhibit highly significant alterations in verbal and visual functionalities, as well as impaired attention and memory. This study was conducted by measuring various outcome measures, including CVLT, COWAT, WMS-LM, and WMS-VR (Ashman et al., 2008). In this comprehensive meta-analysis, 13 case–control studies were included with 1,442 participants to determine the association between severe TBI and cognitive and functional disabilities, including memory, depression, attention deficit, learning disabilities, and verbal and visual impairments. The pooled outcomes of the current analysis revealed that severe TBI has a significant association with altered cognitive and functional abilities. The outcome measuring instruments for the analysis are good in construct validity and reliability, elaborating that these are high-quality measures of primary and secondary outcomes, including depression, verbal, visual, learning, memory, and attention.

A case–control study involving TBI patients has demonstrated that several cognitive dysfunctions were highly significant in patients over the age of 65 years who had experienced severe TBI. This study assessed learning and visual abilities using outcome measures such as WMS-VR and WMS-LM. Outcomes from the study revealed that in older adult patients, significant alterations in visual and learning abilities were found (Breed et al., 2008). Similarly, in younger severe TBI patients aged 6 to 40 years, statistically significant alterations in intellectual, verbal, and memory strengths were found, which were measured using MS-III and WMS-R outcome measures (Mangels et al., 2002; Hawley, 2004). Similarly, a case–control study was conducted in 2004, including 117 participants, consisting of 33 control subjects and 84 severe TBI patients aged 2–7 years. The outcomes revealed that severe TBI patients in this group exhibited impaired learning and memory functionality, which was assessed using the EOWPVT and NMI outcome measures (Anderson et al., 2004).

To prevent cognitive and functional disabilities in severe TBI patients, it is important to get immediate and specialized medical care, with a focus on reducing injury and alleviating cognitive dysfunctionalities. Offering cognitive therapy and emotional support can help to improve their long-term recovery. Further advanced research studies are required in this field to provide a more comprehensive understanding of the association between severe TBI and functional and cognitive disabilities, including depression, attention deficit, verbal and visual impairment, and learning abilities. Novel research studies are needed to explore the intricate mechanisms underlying the association between severe TBI and cognitive disabilities, utilizing sophisticated neuroimaging techniques, longitudinal assessments, and detailed neurobehavioral analyses. Moreover, intervention-focused studies aimed at evaluating personalized therapeutic approaches and their impact on long-term functional outcomes will significantly contribute to improving patient care.

Our findings indicated a strong association between severe TBI and the risk of cognitive and functional disabilities. However, there are some study limitations in this meta-analysis, including randomized controlled studies that are not evaluated with reference to some essential factors, including patients’ age, body mass index, ethnicity, social and demographic statuses, sample size, and co-morbidities.

## 5 Conclusion

Our findings demonstrate a strong association between severe TBI and impaired cognitive and functional abilities. The results indicate significant impairments in memory, verbal and visual disabilities, depression inducement, attention deficits, and learning disabilities among individuals with severe TBI compared to the control group. These outcomes highlight the importance of understanding the long-term effects of TBI on various cognitive and functional domains.

## Data availability statement

The original contributions presented in the study are included in the article/supplementary material, further inquiries can be directed to the corresponding author.

## Author contributions

ZS: Conceptualization, Methodology, Writing – original draft. YJ: Software, Writing – original draft. NM: Software, Writing – original draft. WZ: Formal analysis, Writing – original draft. ZY: Formal analysis, Writing – original draft. SW: Data curation, Writing – original draft. QL: Data curation, Writing – original draft. JR: Conceptualization, Methodology, Supervision, Writing – review & editing.

## Footnotes

^1^ https://www.cochranelibrary.com/

^2^ https://pubmed.ncbi.nlm.nih.gov/

^3^ https://www.embase.com/
